# Strategies and opportunities for promoting bioinformatics in Zimbabwe

**DOI:** 10.1371/journal.pcbi.1006480

**Published:** 2018-11-29

**Authors:** Ryman Shoko, Justen Manasa, Mcebisi Maphosa, Joshua Mbanga, Reagan Mudziwapasi, Victoria Nembaware, Walter T. Sanyika, Tawanda Tinago, Zedias Chikwambi, Cephas Mawere, Alice Matimba, Grace Mugumbate, Jonathan Mufandaedza, Nicola Mulder, Hugh Patterton

**Affiliations:** 1 Department of Biology, School of Natural Science and Mathematics, Chinhoyi University of Technology, Chinhoyi, Zimbabwe; 2 African Institute of Biomedical Science & Technology, Harare, Zimbabwe; 3 Department of Crop and Soil Science, School of Agricultural Sciences, Lupane State University, Lupane, Zimbabwe; 4 Department of Applied Biology and Biochemistry, Faculty of Applied Sciences, National University of Science and Technology, Bulawayo, Zimbabwe; 5 Computational Biology Division, Department of Integrative Biomedical Sciences, Institute of Infectious Disease and Molecular Medicine, Faculty of Health Sciences, University of Cape Town, Cape Town, South Africa; 6 Department of Biotechnology, School of Agricultural Sciences and Technology, Chinhoyi University of Technology, Chinhoyi, Zimbabwe; 7 Department of Biotechnology, School of Industrial Science and Technology, Harare Institute of Technology, Harare, Zimbabwe; 8 Department of Clinical Pharmacology, College of Health Sciences, University of Zimbabwe, Harare, Zimbabwe; 9 Department of Chemistry, School of Natural Science and Mathematics, Chinhoyi University of Technology, Chinhoyi, Zimbabwe; 10 National Biotechnology Authority, Harare, Zimbabwe; 11 Centre for Bioinformatics and Computational Biology, Stellenbosch University, Stellenbosch, South Africa; CPERI, GREECE

## Introduction

The increasing applications of advanced technologies in life sciences are fueling the growth of data from genome sequencing, functional genomics experiments, and macromolecular structure determination. Bioinformatics (sometimes interchangeably used with the term “computational biology”) permits researchers to collect, manage, and sift through these massive data sets and derive scientific insight from them [1,2]. Bioinformatics holds a big promise in addressing many of the problems that are facing humanity today, including human health, agriculture, and the environment [3–8]. Consequently, the demand for skilled scientists with the ability to use information technology to solve life science problems has been rising steadily globally.

Similar to other developing countries in Africa, bioinformatics is slowly gaining popularity among Zimbabwean scientists. In this paper, we review the progress made by Zimbabwean scientists in bioinformatics and propose strategies for boosting bioinformatics capacity in the country. To our knowledge, this work is the first attempt to give a comprehensive report of bioinformatics activities in the country. As such, it is inevitable that our review may not be exhaustive and may fall short of mentioning or acknowledging groups or scientists who have contributed or presented their work on other platforms.

## Overview of bioinformatics in Africa

The establishment of the South African National Bioinformatics Institute in South Africa in the 1990s heralded the development of bioinformatics on the continent [9]. Countries such as Kenya and Nigeria established pockets of high-quality bioinformatics teams soon after. However, most African research institutions still lagged behind. The introduction of bioinformatics to the rest of the African continent was slowed down by several challenges that include limited scope of research encompassing bioinformatics-driven objectives, shortage of qualified bioinformatics experts, poor access to powerful computer systems, lack of high-speed internet, poor access to essential databases and software programs, and unreliable power supply [10,11].

Recent funding investments toward large-scale research projects, training, and infrastructure support are helping address the bioinformatics disparities between countries within the continent through establishment of world-class resources and training [12]. The establishment of the African Society for Bioinformatics and Computational Biology (ASBCB) (http://www.asbcb.org) during a World Health Organization/Tropical Disease Research workshop in February 2004 led to a sustainable network of researchers across the continent. A noteworthy initiative with its foundation in the ASBCB is the Human Heredity and Health in Africa Bioinformatics Network H3ABioNet network, whose mandate is to provide bioinformatics support for the Human Heredity and Health in Africa (H3Africa) initiative and to develop bioinformatics capacity across Africa through funding provided by the National Institutes of Health [9,13]. H3ABioNet has focused on building infrastructure and implementing tools that enable collaborations and data transfer across the resource-limited continent. Since its conception, H3ABioNet has been capturing and measuring metrics across the consortium using the Network Capacity Database, a relational database [14], and it is anticipated that analysis of these data will give insight into the impact of the H3ABioNet consortium.

In order to speed up the development of human capital in bioinformatics in Africa, various bioinformatics training initiatives happening across Africa provide training via face to face short courses and other online-based initiatives. Approaches have been suggested such as train-the-trainer approach, or placements and/or visits from experts for knowledge transfer purposes into specific research institutes and/or groups [15]. Also, the H3ABioNet consortium has recently established an education committee tasked with developing curriculum guidelines for bioinformatics training in Africa [16]. Such training initiatives are worth exploring or leveraging on for any new bioinformatics initiative. Recently, three universities and four research institutes in Kenya, Tanzania, and Uganda collaborated to create The Eastern Africa Network for Bioinformatics Training (EANBiT), whose aim is to develop a critical mass of practitioners who can develop and use bioinformatics approaches to biosciences. EANBiT is supported by the Fogarty International Center of the National Institutes of Health under award number U2RTW010677 and institutional partners in the network.

## Bioinformatics in Zimbabwe

**Fig 1:**
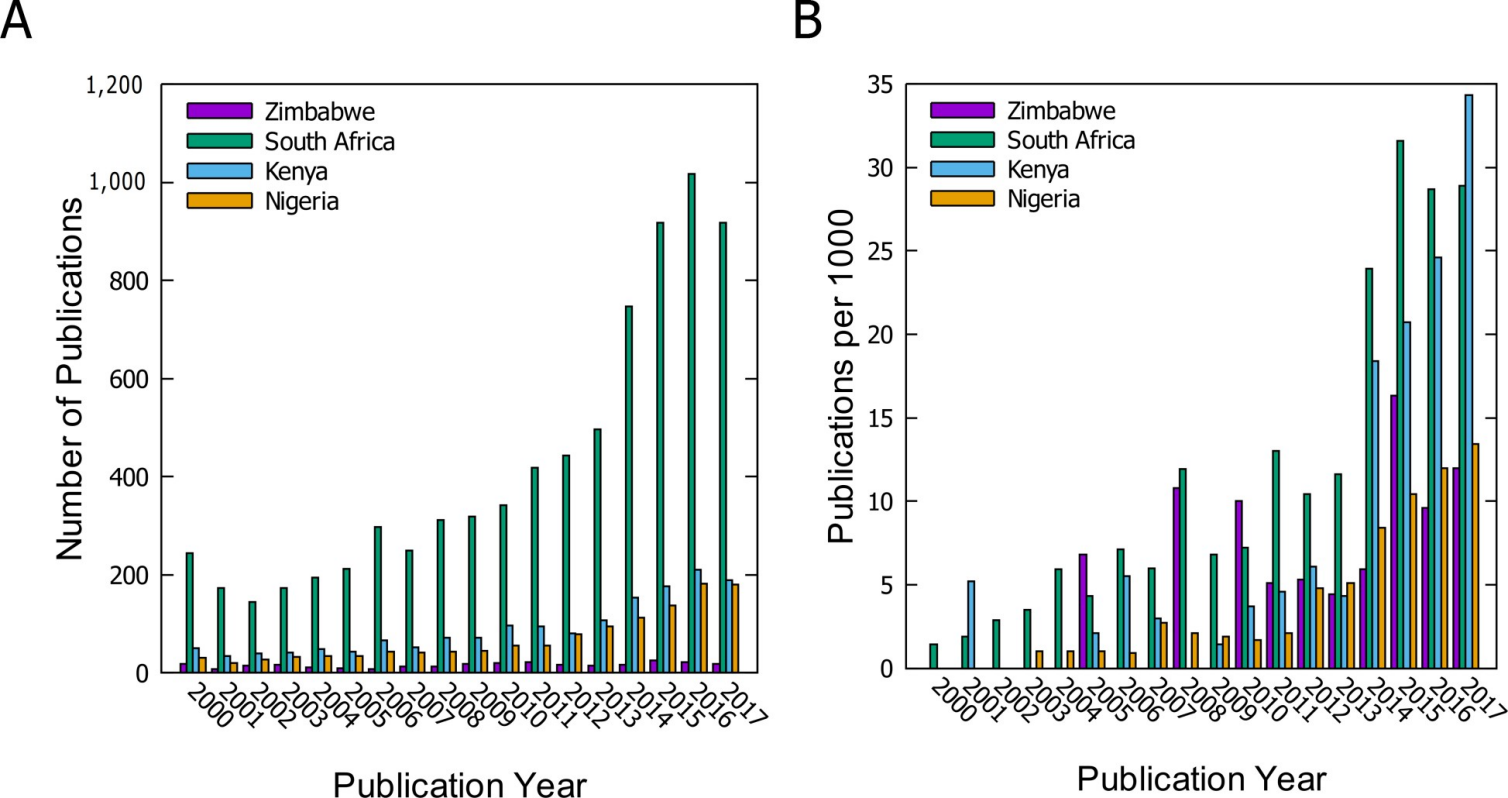
Bioinformatics publications authored by scientists affiliated with Zimbabwean institutions compared to South Africa, Kenya, and Nigeria. (A) The absolute number of bioinformatics papers. (B) The number of bioinformatics papers normalized to the total outputs in the life sciences. A python script was used to obtain peer-reviewed research articles published between 2000 and 2017 and indexed in PubMed.

The prospects for a vibrant bioinformatics community in Zimbabwe are excellent, given the outstanding need for life science research and the country’s rich diversity of resources from human populations, crops, animal species, and the environment. Despite this potential, the application of bioinformatics in Zimbabwe lags behind some African countries, such as South Africa, Kenya, and Nigeria, as indicated by the low national publication record (see Fig 1A and 1B). Fig 1A shows the number of peer-reviewed publications in bioinformatics-related areas with at least one author affiliated with a Zimbabwean institution. The data clearly indicate that the absolute output from Zimbabwe is significantly less than other select African countries (South Africa, Kenya, and Nigeria). However, bioinformatics as a research field producing peer-reviewed articles is not completely absent from the Zimbabwean research landscape. Fig 1B, which shows the output of bioinformatics papers normalized to the total output in the life sciences from the same select African countries, shows that bioinformatics research as a fraction of total research in life sciences is comparable to that of Nigeria. This figure makes the important point that, although the total bioinformatics research activity is low, it forms a significant proportion of research in the life sciences. This available bioinformatics research provides an extant research kernel that can be grown.

There are some major developments in the country that promise to spur the development of bioinformatics in Zimbabwe. These are, inter alia, the following:

- In 2015, the government of Zimbabwe commissioned a high performance computer cluster that is housed at the University of Zimbabwe (UZ) (http://www.zchpc.ac.zw/). The cluster has a theoretical computing capacity of up to 36 Tflops.
- In 2016, the National Biotechnology Authority of Zimbabwe (NBA), the University of Mauritius, Chinhoyi University of Technology (CUT), and Harare Institute of Technology (HIT) organized the first Zimbabwe Bioinformatics Symposium. The symposium, which was held at the HIT campus, was attended by academics and students from the country's universities and research institutes as well as some international collaborators.
- Starting in September 2017, the African Institute of Biomedical Science and Technology (AiBST) has hosted the first Zimbabwean bioinformatics node as part of the H3Africa, H3ABioNet V2.

### Bioinformatics in human health

Zimbabwe, like most African countries, battles with serious health challenges, particularly in infectious diseases such as the human immunodeficiency virus/acquired immunodeficiency syndrome (HIV/AIDS), malaria, and tuberculosis (TB) [17–20]. In addition, noncommunicable diseases are on the rise and contribute significantly to comorbidities. Researchers in Zimbabwe have started employing bioinformatics techniques to help unravel the genetic and environmental determinants for these diseases.

To better understand the HIV dynamics in vertical transmission, some research groups explored the diversity of HIV sequences from infected infants and various compartments from their mothers. In one study, genotypic analysis of HIV-1 envelope third variable loop sequences of infected but drug-naive women during pregnancy and their infected infants was carried out to predict virus coreceptor utilization in vertical transmission [21]. Using similar methods, other groups investigated the viral characteristics associated with high-risk genotypes of human papillomavirus that are associated with malignancy [22]. These studies represent an effort to use bioinformatics to produce data of clinical relevance for Zimbabwe and beyond.

Zimbabwean researchers have been at the forefront of developing the field of pharmacogenomics in Africa [23–26]. Spearheaded by the Departments of Biochemistry and Clinical Pharmacology at the UZ and AiBST, pharmacogenomics research has mostly focused on understanding the role of human and pathogen genetic variation in drug response, side effects, and therapeutic outcomes in the management of HIV, TB, and malaria. One of the most important outputs from these efforts has been the establishment of a biobank and a pharmacogenetic database for African populations, a resource developed to provide baseline understanding of pharmacogenetic variation for potential application toward tailoring drug treatment [24]. Further work has enabled elucidation of key markers, which are prevalent in African populations and are associated with side effects of the anti-retroviral drug efavirenz [25,26].

Zimbabwean scientists have started to embrace computational methods for the discovery of drug targets and for the identification of new disease markers to improve early diagnosis or develop new therapeutic strategies. For example, a study at AiBST used in vitro and in silico approaches to show that thiabendazole is a potent mechanism-based inhibitor of cytochrome P450 1A2 [27]. In the study, potential drug–drug interactions were also simulated. These and other simulation efforts are likely to increase given the workshops on computer aided drug discovery (CADD) conducted at the Zimbabwe Centre for High-Performance Computing (ZCHPC) in 2016.

Local scientists have not actively pursued high-throughput functional genomics approaches such as proteomic profiling for mechanism-based drug discovery and drug repurposing. This is despite the fact that gas chromatography–mass spectrometers (GC-MS) and liquid chromatography–mass spectrometers (LC-MS) can be found at research institutions such as AiBST, National Microbiology Reference Laboratory, and Standards Association of Zimbabwe Laboratories. This is mainly because there are few researchers with sufficient knowledge of proteomics (or other functional genomics approaches such as transcriptomics and metabolomics) to carry out research encompassing “omics”-driven objectives. Also, the organizations with the GC-MS and LC-MS generally do not have technicians with expertise in proteomics, and thus there are no optimized protocols and data analysis pipelines.

### Bioinformatics in food security

Zimbabwe produces a large variety of agricultural products meant for food, such as maize, sorghum, groundnuts, fruit, and vegetables, in addition to livestock and poultry. Generally, bioinformatics can be used to efficiently gain access to “omics” data available in the publicly available repositories and to make available the data to research scientists involved in animal or crop breeding. For example, local scientists can employ genomics analysis to aid in the assessment of genetic diversity and identification of important genetic traits for the production of crops and livestock with desirable traits including high yield and disease resistance [7,8]. However, there is little bioinformatics activity in that direction in Zimbabwe. Research institutions such as universities, the Scientific and Industrial Research and Development Centre, the International Maize and Wheat Improvement Centre (CIMMYT), and the International Crops Research Institute for the Semi-Arid Tropics, are, however, well positioned to adopt bioinformatics techniques owing to the regional and international collaborative nature of their research projects. The following examples illustrate how such collaborations are important.

1. Researchers at CIMMYT, working with their international collaborators, conducted a metabolomic profiling study of maize leaves to investigate the effects of drought, heat, and combined stress on grain yield in field crops [28]. This kind of work is important in the development of abiotic stress-resistant and/or tolerant cultivars, thereby improving food security.
2. In the livestock sector, a genome-wide association study of the Zimbabwean goat, pigs, and cattle has been done by the South African Biotechnology Research Institute under the Agricultural Research Council in collaboration with the UZ [29].

### Metagenomics studies

Some metagenomic efforts are being made at some universities such as CUT and National University of Science and Technology (NUST), with the aim of improving environmental sustainability and the bioprocessing industry. Among other works, the NUST group presented the whole-genome shotgun metagenome sequences of the greater kudu (*Tragelaphus strepsiceros*) rumen digesta revealing its diverse microbial community and some novel hydrolytic enzymes [30]. While all the preliminary wet laboratory procedures, including the metagenomic DNA extraction, were carried out at NUST, subsequent steps were carried out at international institutes. Whole-genome sequencing of the metagenomic DNA was performed at Inqaba Biotech Laboratories in Pretoria, South Africa, and the sequences were analyzed at the European Bioinformatics Institute of the European Molecular Biology Laboratory. In addition, a research group at CUT, working with South African collaborators, performed functional screening of the metagenome of the hindgut bacterial symbionts of a termite, *Trinervitermes trinervoides*, to discover open reading frames for 25 cellulases and hemicellulases [31]. These metagenomic efforts in the country have biotechnological significance because they may lead to, among other things, the isolation of novel enzymes such as cellulases, which can be used in the production of biofuels.

### Bioinformatics education

#### University programs

Zimbabwe has no institution offering dedicated bioinformatics degrees at the undergraduate or graduate level. However, relatively new universities (CUT, HIT, Lupane State University [LSU]) have integrated bioinformatics courses in their undergraduate biotechnology and/or biological sciences degree programs. Given the importance of bioinformatics, it would be desirable if all universities offering undergraduate degrees in the life sciences introduced bioinformatics courses. This will help create a new skilled workforce, produce more tangible results in a shorter period of time, and provide a strong foundation for the development of specialized MSc-and PhD-level bioinformatics programs. It is encouraging to note that some universities offer Master’s and PhD research projects in which bioinformatics-driven objectives form a significant component, e.g., projects involving genomic, proteomic, and metabolite profiling.

Most early career scientists teaching at universities are molecular biologists who hold PhDs from overseas universities or from other countries in Africa (mainly South Africa). This situation has complicated the true assessment of local skills needs and can have a disruptive effect on development of bioinformatics in the country. On the other hand, the experienced generation of molecular biologists at most universities was trained before the widespread application of bioinformatics in research. These biologists also need training in this area for them to teach bioinformatics to their students.

#### Short courses

Since 2005, several capacity-building initiatives are ongoing and are facilitated by the Research Council of Zimbabwe, Biomedical Research and Training Institute, National Biotechnology Authority (NBA), and AiBST. These capacity-building activities are presented through symposia and short course training. The bioinformatics topics covered in these courses have included genome browsing, DNA sequence analysis, and DNA barcoding. These short courses have been complemented by several exchange programs with international partners such as Stanford University, University of Oslo, University of Cape Town, and Biodiversity Institute of South Africa, which supported some students in their MSc and PhD programs in molecular-biology–related disciplines. In August 2016, workshops were conducted in which participants from the country's universities and major research institutions were trained in computational chemistry and CADD at the ZCHPC. The training focused on software for drug discovery such as GROMACS, Schrodinger Suite including Maestro, and molecular visualization tools like Pymol and Chimera.

### Scientific networking and collaborations

Networking among local scientists presents potential opportunities for peer support, collaborative research, and effective postgraduate training of students among bioinformatics researchers. One of the reasons for poor networking among Zimbabwean scientists is that some institutions do not have comprehensive websites, and potential collaborators find it difficult to identify groups to work with. This puts many as yet unknown early career scientists at a disadvantage. We have summarized (Table 1) the institutions pursuing bioinformatics-related research. However, most of the research findings from early career scientists are yet to be published in peer-reviewed journals. We hope that publishing this list will encourage networking and collaborations among local scientists, as well as with the international community.

**Table 1:** Research activities currently underway in Zimbabwean institutions.

Institution	Research areas	Official website
AiBST	**•** Medical genomics of HIV and TB drug resistance **•** Pharmacogenomics studies **•** H3ABioNet Node	www.aibst.com
CUT	**•** Identification of new anti-infective agents and drug targets using chemogenomics and molecular modeling **•** Systems biology and proteomic approaches to understand molecular mechanisms of plant stress tolerance **•** Bioprospecting for novel hydrolytic enzymes from animals and natural environments using metagenomics approaches **•** Metabolomic and genomic profiling toward cultivation strategies of *Fadogia ancylantha*	www.cut.ac.zw
HIT	**•** CADD	www.hit.ac.zw
LSU	**•** Application of bioinformatics for crop improvement **•** Phylogenetic studies of wild edible mushrooms of Zimbabwe	www.lsu.ac.zw
NBA	**•** Bioinformatics for toxicological risk assessment of potential allergens resulting from use of new and emerging technologies such as synthetic biology, new plant breeding technologies (precision breeding technologies, nanobiotechnology)	www.nba.ac.zw
NUST	**•** Phylogenetic studies of viruses of medicinal and veterinary importance **•** Bioprospecting for novel hydrolytic enzymes from animals and natural environments using metagenomics approaches	www.nust.ac.zw
UZ	**•** CADD **•** Pharmacogenomics	www.uz.ac.zw
ZCHPC	**•** HPC training **•** Supercomputing facility	www.zchpc.ac.zw

**Abbreviations:** AiBST, African Institute of Biomedical Science and Technology; CADD, computer-aided drug discovery; CUT, Chinhoyi University of Technology; H3ABioNet, Human Heredity and Health in Africa Bioinformatics Network; HIT, Harare Institute of Technology; HIV, human immunodeficiency virus; HPC, high-performance computing; LSU, Lupane State University; NBA, National Biotechnology Authority of Zimbabwe; NUST, National University of Science and Technology; TB, tuberculosis; UZ, University of Zimbabwe; ZCHPC, Zimbabwe Centre for High-Performance Computing.

Following the first Zimbabwe Bioinformatics Symposium, the NBA made efforts to establish the Bioinformatics Consortium of Zimbabwe (BCZ), which aimed at (1) continually promoting networking among bioinformaticians, (2) promoting the exchange of ideas and resources, and (3) facilitating local and international collaborations. Through this consortium, an application was submitted and accepted for the Human Health Node through the H3ABioNet. With this encouraging development, there is a great opportunity for increased networking and membership of the BCZ.

We note that only a handful of researchers from Zimbabwe have joined the International Society for Computational Biology (ISCB) (https://www.iscb.org) or ASBCB. These societies provide an excellent forum for the interaction of researchers in the area of bioinformatics, which is highly beneficial to the members. Nonparticipation in the ISCB and ASBCB is probably due to the slow development of bioinformatics research in the country coupled with poor funding opportunities for local researchers.

## Perspectives and conclusion

### Proposed strategy for implementing bioinformatics at national level

**Fig 2:**
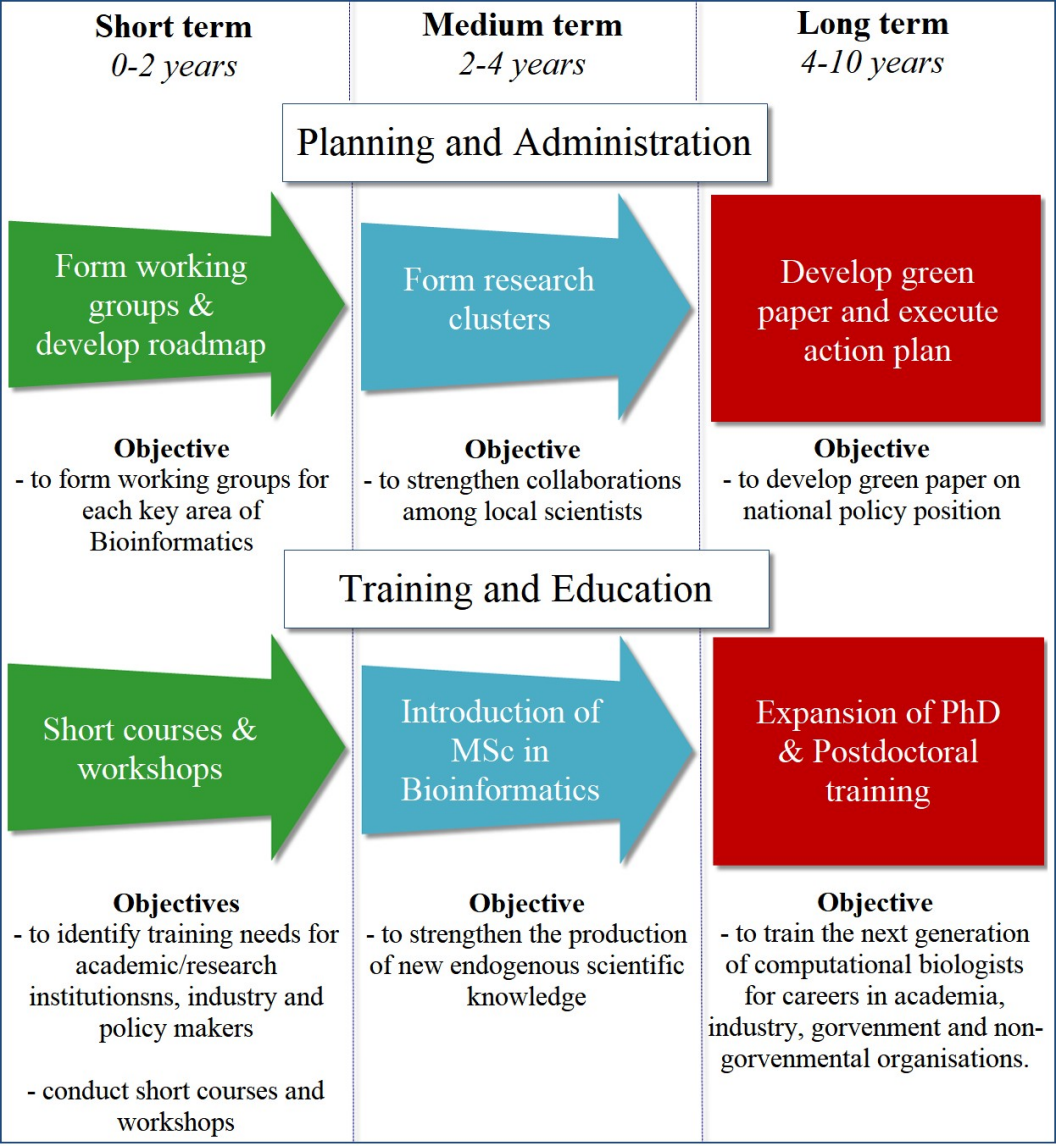
Proposed strategies for promoting bioinformatics in Zimbabwe. In the short and medium terms, formation of research groups and clusters is essential while working toward the development of a green paper in the long run. In education and/or training, focus should be placed on workshops in the short term followed by introduction of MSc and PhD programs in the medium and long term. See main text for details.

We are confident that a clear national road map aimed at developing independent and dedicated research and support structures as demonstrated by the South African experience [9] will accelerate implementation of bioinformatics research in Zimbabwe. Therefore, we propose that we devise strategies as summarized in Fig 2.

### Short-term strategies

In the short term, the BCZ should call a meeting of all Zimbabwean bioinformatics researchers, educators, industry leaders, and government policy makers to discuss bioinformatics resources and opportunities. Such a situation would allow for the identification of national priorities for bioinformatics research. The specific research areas chosen should be in line with the developmental needs of the country and be feasible in terms of the country’s resources. Although there was an interaction of local researchers in the first Zimbabwe Bioinformatics Symposium held in Harare in 2016, the major focus was to present past and present research results by local and international scientists. The BCZ, which should adopt a more transparent membership recruitment policy, should drive the development of a roadmap and would be expected to review progress at least annually through research symposia. In that regard, the BCZ needs to develop a clear administration structure that should be tasked with coming up with a constitution and a website to facilitate the creation and/or editing of profiles of bioinformatics research groups, thus helping to publicize bioinformatics initiatives in Zimbabwe. In addition, the BCZ would be expected to provide ongoing support to members through web-based portals.

During the development of the national roadmap, an International Advisory Committee (IAC) needs to be formed, which would be responsible for providing guidance on the planning and implementation of the project as a whole. The IAC should include members from the region and the international community—thus combining the knowledge of the peculiarities in the region and the more objective points of view from the international community. For the IAC to be effective, it should be composed of members with no conflicts of interest regarding the development of bioinformatics in Zimbabwe.

To address the critical skills shortage, the short-term strategy would be giving focus to seminars, conferences, and workshops that are coordinated at a national level and tailored to meet the training requirements of postgraduate students, academics, and relevant industry players. Given that bioinformatics is an interdisciplinary field, postgraduate students and researchers can be drawn from a broad range of disciplines including biology, chemistry, statistics, and computer science. For beginners, the courses may be designed to teach participants important skills such as coding for biology, biological databases, data mining, etc. More advanced topics—including systems biology, proteomics, metabolomics, and structural bioinformatics—should be considered for those who are already familiar with the basic introductory topics. Prioritizing short courses at this stage will be important to ensure that research students become acquainted with the latest advancements in bioinformatics and thus design their research projects accordingly. Involvement of the government and industry is crucial because these can help provide postgraduate students funding for attending international conferences and training events that could be a good means of quickly getting the bioinformatics community active. Mentoring and training of trainers will be crucial for building capacity and developing critical mass of individuals with bioinformatics skills. In addition, the BCZ needs to develop an online platform for sharing educational and/or training material, code, and bioinformatics protocols. These measures will ensure easy access to information and speed up developments in the field.

### Medium-term strategies

Once the stage has been set for the national coordination of bioinformatics activities, these need to be strengthened by the formation of research clusters. Because bioinformatics is inherently interdisciplinary in nature, involvement of computer scientists, statisticians, etc., is crucial. Formation of multidisciplinary research clusters should make it easier to procure research funding from local sources and competitive international sources. Research clusters should be supported at a national level by coordinating and facilitating access to infrastructure facilities. At present, many local research groups work in isolation, and some are not aware of where to access resources locally, thus contributing to a poor publication record. Ongoing research programs such as those highlighted in Table 1 could be used as the basis for development of specific bioinformatics skills.

It can be argued that the establishment of an MSc (or equivalent postgraduate qualification) in bioinformatics is urgent in Zimbabwe. One way to do this, as a starting point, would be to identify one university and support it to set up a national bioinformatics training and resource institute. The institute could house the majority of bioinformaticians available in the country and be key in the training of both students and staff and help in local bioinformatics research activities. The institute will also work closely with the BCZ to make sure that no researcher and/or research is excluded and unrecognized. It is refreshing to note that CUT has started efforts to draft regulations for MSc Bioinformatics degree programs by coursework in collaboration with H3ABioNet. Associating with H3ABioNet is ideal because it helps one benefit from the experience of setting up an MSc Bioinformatics program in an African setting to address African needs. Also, it helps to address the skills shortage for lecturers because the H3ABioNet has coordination strategies for sharing human resources from distant universities, including via online education. CUT has also signed a memorandum of understanding with AiBST in an arrangement that would see CUT offering an MSc in Genomics and Precision Medicine starting in 2019.

### Long-term strategies

In the long term, it should be possible that a national green paper on bioinformatics be produced and agreed policies implemented. This can be achieved by involving the government from an early stage in defining the priorities for support. For example, given the burden of both communicable and noncommunicable disease in Africa, all stakeholders could agree that health is a national priority for bioinformatics support. Tackling emerging risks of viral infections and drug resistance can be addressed by applying bioinformatics to understand pathogen genomes, to follow resistance patterns, and for identifying drug targets.

With the background work done, the culmination of education and training efforts should see significant registrations for PhD studies and postdoctoral training. The importance of doctoral-level training at a local level for any economy need not be over-emphasized. Locally trained graduates would help tackle local problems more effectively, either during their training or after graduation. Indeed, the publication record for the country would significantly improve. The government can play an important role in supporting the PhD studies financially.

### Conclusion

Despite limited funding sources, we find that some bioinformatics research efforts are being made by several groups involved in biomedical research, CADD, agriculture, and biotechnology. However, the most active areas are those involved in human health. Most of the biomedical research in Zimbabwe is being done by a few well-established institutions with links and partnerships to established institutions in the region or overseas. The research focuses mainly on the primary diseases of poverty (TB, malaria, HIV/AIDS, and other opportunistic pathogens). However, the national publication record is still low when compared to some African countries that have embraced bioinformatics, mainly due to poor funding, poor networking, and inadequate training opportunities. Solving these problems would require coordination at a national level.

We have proposed strategies for stimulating increased activities in bioinformatics in Zimbabwe. These strategies include the recognition of the BCZ that was established in 2016 to lead the formulation and implementation of the roadmap to bioinformatics growth and coordinate national activities, among other goals. In order for the BCZ to be more viable internationally, it should be affiliated with international platforms such as the ISCB and the ASBCB. Clearly, the envisaged responsibilities of the BCZ are serious and require an effective administrative structure. An effective BCZ is important because it can aid government policy makers in mapping the direction of science and technology research in the country.

For a sustainable implementation of bioinformatics in the country, we suggest that the country aim to become a data producer and bioinformatics service provider on a specific and firm set of limited domains rather than being an unfocused bioinformatics user in a broad range of disparate fields. Therefore, at this stage, the country should aim to operate within a targeted node in a pan-African network. In our view, although international linkages are important, the commitment to bioinformatics capacity development by the government and industry players would be key to the success of the bioinformatics endeavors in the country.
